# Dolphin-inspired adaptive coordination for online multi-robot path planning in dynamic environments

**DOI:** 10.3389/fnbot.2026.1993109

**Published:** 2026-10-29

**Authors:** Meijun Liu, Haibin Wu, Lei Qin, Zetong Lu, Rui Cheng, Lijuan Chen

**Affiliations:** 1 School of Automation, Harbin University of Science and Technology, Harbin, China; 2 School of Measurement-Control Technology and Communications Engineering, Harbin University of Science and Technology, Harbin, China; 3 Guangdong Huibo Robotics Technology Co., Ltd., Foshan, China

**Keywords:** adaptive operator selection, bio-inspired optimization, differential evolution, dynamic obstacle avoidance, embodied autonomous systems, multi-robot path planning

## Abstract

**Introduction:**

Online multi-robot path planning requires repeated joint motion decisions amid static obstacles, moving obstacles, and other robots.

**Methods:**

This study introduces the Dolphin Cooperative Foraging Optimizer (DCFO), a population-based method that adapts its search strategy within each replanning call. DCFO combines an elite-guided operator with three differential evolution operators. Historical operator-feedback counts determine selection probabilities; a stage-dependent rule determines which selected operator is executed; and population size decreases with the nominal evaluation budget. A masked, regularized inverse covariance perturbation is triggered when all population fitness values coincide. Search performance was evaluated on the CEC2020 and CEC2022 benchmark suites, followed by MATLAB simulations with 6 and 12 robots.

**Results:**

Compared with four population-based planners and the Dynamic Window Approach, DCFO achieved the shortest aggregate path in both tested scenarios. Path length was 2.74% shorter than the next-best result in the 6-robot scenario and 8.30% shorter in the 12-robot scenario. DCFO also recorded the lowest average simulation execution time in the 12-robot scenario, although it was not the fastest method in the 6-robot scenario.

**Discussion:**

These results identify adaptive operator portfolios as a promising approach to joint motion-command generation in dynamic multi-robot simulations. Future research will extend the framework to physical robot platforms and more diverse operating conditions.

## 1 Introduction

Mobile robots repeatedly convert information about their surroundings into motion, which changes the conditions for the next decision. This coupling between the body, environment, and control motivates research on embodied autonomy (Sirithunge et al., 2024). In a robot team, each robot must also account for the motion of its neighbors. Online multi-robot path planning, therefore, requires coordinated decisions that promote goal progress while responding to changing collision risks (Qin et al., 2023; Zhang et al., 2024; Wu and Li, 2026).

Graph search, sampling-based, reactive, and optimization-based methods offer different ways to construct such decisions (Qin et al., 2023). A joint command formulation makes interactions between robots explicit; however, its dimension grows with team size, and threshold-based collision penalties can make the objective discontinuous. Population-based methods can evaluate these objectives without the use of derivatives, although this results in repeated candidate evaluations (Salgotra et al., 2024; Zhao et al., 2025). Akay and Yildirim (2023) demonstrated the use of multiple adaptive sine–cosine and differential strategies for online multi-robot planning, providing a relevant basis for investigating how search operators should be allocated within a limited replanning budget.

An operator portfolio can combine search mechanisms with different responses to population geometry and search progress. For example, the Snow Ablation Optimizer (SAO) uses stochastic updates guided by elite solutions (Deng and Liu, 2023). Within differential evolution (DE), differential evolution based on covariance matrix learning and bimodal distribution parameter setting (CoBiDE) uses covariance learning and bimodal parameter distributions (Wang et al., 2014). Individual-dependent differential evolution (IDE) adapts variation to individual rank and search stage (Bujok and Tvrdik, 2017), and jSO couples current-to-pbest mutation with parameter memories and population reduction (Brest et al., 2017). Recent studies on adaptive operator selection also investigate feedback-driven allocation of search effort among heterogeneous operators (Yin and Xiang, 2024). These methods motivate an integrated portfolio whose numerical search behavior and performance within a robot control loop can be evaluated together.

This study addresses two related design questions: How to allocate a finite replanning budget among heterogeneous operators and how to resume search when the population has identical fitness values. Operator-specific acceptance and improvement counts provide feedback for the first question, while population history provides geometric information for the second. The resulting design is evaluated through numerical benchmarks and robot-level simulation outcomes.

We propose the Dolphin Cooperative Foraging Optimizer (DCFO) to investigate these questions. Dolphin foraging serves as a design analogy for coordinated herding, heterogeneous individual actions, and feedback-driven redirection; the method is not intended to reproduce dolphin cognition or neural processing. The contribution integrates an elite-guided update and three established differential evolution structures with feedback-based operator selection, stage-dependent remapping, population reduction, and stagnation recovery. Evaluation combines numerical benchmarks, an internal selection policy comparison, and two dynamic multi-robot simulations. The robot experiments compare aggregate path length, movement steps, recorded execution time, and accumulated objective cost, focusing on trade-offs rather than a single ranking.

## 2 Materials and methods

### 2.1 Online multi-robot planning problem

At each replanning step, a candidate command for *M* robots is written as *x* = (*v*_1_, *u*_1_, …, *v*_*M*_, *u*_*M*_), with a decision dimension of *D* = 2*M*. The variable *v*_*i*_ is the commanded displacement of robot *i* during one planning step, and *u*_*i*_ is an encoded heading coordinate. In this implementation, both variables are bounded by *b*_*min*_ = 1 and *b*_*max*_ = 1.5, and the physical heading is obtained from Equation 1:


$$\begin{array}{l} \theta_{i}=2\pi \frac{u_{i}-b_{min}}{b_{max}-b_{min}} \end{array}$$ (1)

Thus, 0 ≤ θ_*i*_ ≤ 2π. If $p_{i}={\left[p_{i,x},p_{i,y}\right]}^{T}$ is the current position, the one-step position $p_{i}^{+}$ is given by Equation 2:


$$\begin{array}{l} p_{i}^{+}=p_{i}+v_{i}\left[\begin{array}{l} \cos\theta_{i}\\ \sin\theta_{i} \end{array}\right] \end{array}$$ (2)

Here, the distance is measured in centimeters, and *v*_*i*_ is the distance commanded during one discrete planning step, rather than a continuous-time velocity. Following the simulation model proposed by Akay and Yildirim (2023), a candidate is evaluated using Equation 3:


$$\begin{array}{l} J=\sum_{i=1}^{M}\left(||p_{i}^{+}-p_{i}|{|}_{2}+||p_{i}^{+}-g_{i}|{|}_{2}+\kappa [C_{i,s}+C_{i,d}+C_{i,r}]\right) \end{array}\begin{array}{l} \kappa =10^{4} \end{array}$$ (3)

In this expression, *J* is the total one-step cost, *g*_*i*_ is the goal of the robot *i*, and $||a|{|}_{2}=\sqrt{a^{T}a}$ denotes the Euclidean norm. The non-negative integers *C*_*i, s*_, *C*_*i, d*_, and *C*_*i, r*_ count threshold violations involving static obstacles, dynamic obstacles, and other robots, respectively. Static and dynamic clearances are calculated by adding the obstacle radius to twice the robot radius, while the inter-robot threshold is set at three robot radii. The large penalty κ strongly penalizes violations relative to short-distance gains, but it does not impose a hard safety constraint. Clearances are evaluated between each proposed robot position and the current positions of obstacles or neighboring robots. The outer loop applies motion updates only to robots more than 1 cm from their goals, updates the scene accordingly, and replans until all robots satisfy this tolerance.

### 2.2 DCFO planning framework

The DCFO organizes multi-robot coordination as a closed-loop sequence of state acquisition, joint command optimization, and scene updates (Figure 1). At each planning step, the simulator supplies current robot positions, obstacle states, and goal locations. Candidate displacement and heading commands are evaluated using the one-step objective defined in Section 2.1. The optimizer selects a joint command, the simulation applies it to the active robots, and the updated scene provides the state for the next planning step.

**Figure 1:**
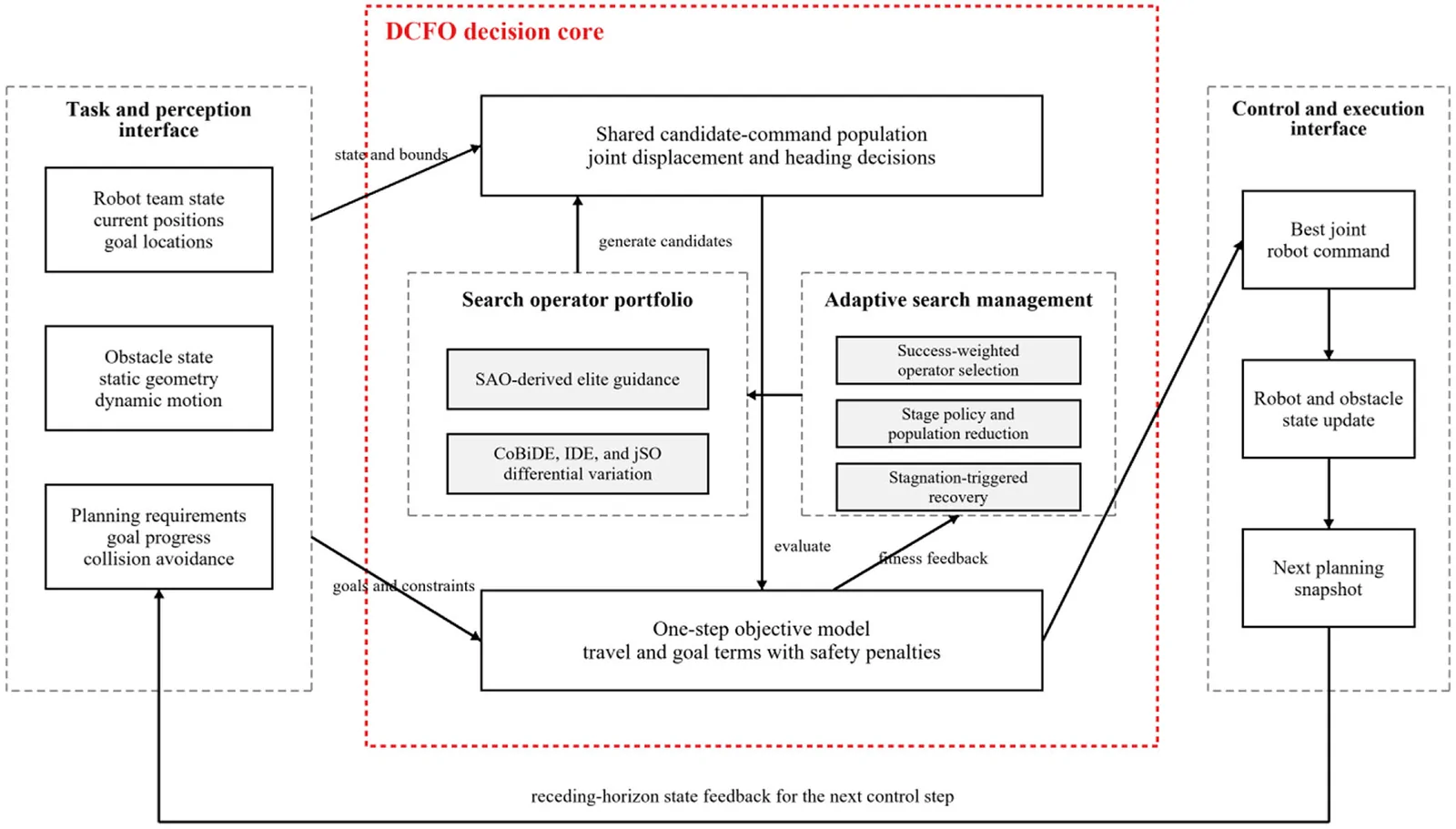
Functional architecture of the closed-loop DCFO planner. The simulator supplies robot positions, obstacle states, goals, and planning constraints. A shared population connects the search operators, adaptive selection, and one-step objective. The selected command updates the simulated robot states. Arrows indicate information exchange. The label “success-weighted” refers to the operator-specific feedback in Section 2.3.3, not a uniform acceptance count or the escape-event rate in Table 2.

Within the optimizer, elite guidance and differential evolution operators generate candidate commands from a shared population. Historical operator feedback counts guide operator selection, while the stage policy, population reduction, and covariance-based perturbation regulate the search within each replanning call. Cooperative herding, heterogeneous individual foraging, and feedback-driven redirection provide the behavioral analogies for these mechanisms (Table 1). The framework uses simulated state information and does not implement a neural or sensory model.

**Table 1:** Behavioral inspiration and computational realization of the DCFO.

Behavioral abstraction	Implemented mechanism	Computational role
Cooperative herding	Elite-guided SAO-derived update	Concentrates candidates around high-quality command regions while retaining stochastic variation.
Heterogeneous pursuit	Mutation portfolio related to CoBiDE, IDE, and jSO	Provides complementary exploration and refinement behaviors within one replanning budget.
Experience-dependent choice	Four-operator feedback-based roulette with stage-dependent remapping	Weights sampling by operator-specific acceptance or strict-improvement counts and redirects selected operators according to search progress.
Feedback-based redirection	Masked, regularized covariance-guided perturbation	Reorients a fully stagnant population using the geometry of retained solutions and a periodic amplitude factor.

### 2.3 Dolphin cooperative foraging optimizer

In the optimizer description, the index *i* identifies a candidate solution rather than a robot. Let *N* denote the population size and *D* denote the solution dimension. Component *x*_*ij*_ of candidate *i* is initialized using Equation 4:


$$\begin{array}{l} x_{ij}=lb_{j}+r_{ij}\left(ub_{j}-lb_{j}\right) \end{array}$$ (4)

where *i* = 1, 2, …, *N*, *j* = 1, 2, …, *D*, *lb*_*j*_ and *ub*_*j*_ are the lower and upper bounds of dimension *j*, and *r*_*ij*_~*U*(0, 1) are independent uniform samples. The DCFO maintains four candidate generation operators: one SAO-derived elite operator and three operators related to CoBiDE, IDE, and jSO. At each generation, feedback-based roulette selection chooses one operator for the population, subject to the stage policy below. A trial replaces its parent when its objective value is no greater than the parent's value. Thus, an accepted trial includes an equal-cost replacement and does not necessarily represent strict improvement.

The replacement rule and the feedback-counting rule are not identical. Section 2.3.3 specifies which accepted trials contribute to each operator's feedback counter.

Search progress is represented by the normalized evaluation ratio ρ defined in Equation 5:


$$\begin{array}{l} \rho =\frac{FES}{MFES} \end{array}$$ (5)

where *FES* is the nominal number of function evaluations already counted and *MFES* is the nominal evaluation budget.

#### 2.3.1 Cooperative elite-guided operator

Candidates are ranked by increasing cost. Let *e*_1_, *e*_2_, and *e*_3_ be the three best candidates, and let *e*_4_ be the component-wise mean of the best half of the population. For candidates assigned to the exploratory branch, component *j* is generated using Equation 6:


$$\begin{array}{l} x_{ij}^{\prime }=e_{k_{i},j}+z_{ij}\left[\alpha \left(e_{1,j}-x_{ij}\right)+\left(1-\alpha \right)\left(e_{4,j}-x_{ij}\right)\right] \end{array}$$ (6)

where $x_{ij}^{\prime }$ is component *j* of the trial generated for candidate *i*, *k*_*i*_ is sampled uniformly from {1, 2, 3, 4}, *z*_*ij*_~*N*(0, 1), with α defined in Equation 7:


$$\begin{array}{l} \alpha =0.2+0.6\rho \end{array}$$ (7)

The alternative elite-based branch uses Equation 8:

$$\begin{array}{l} x_{ij}^{\prime }=fe_{1,j}+z_{ij}\left(e_{1,j}-x_{ij}\right) \end{array}$$ (8)

where the stage-dependent coefficient *f* is defined in Equation 9:


$$\begin{array}{l} f=0.3\left(1-\rho \right) \end{array}$$ (9)

In both branches, independently sampled standard normal variables provide stochastic variation. Boundary reflection is applied before fitness evaluation.

#### 2.3.2 Differential evolution operators

The remaining three portfolio members use differential evolution mutation and crossover. The CoBiDE-related operator first forms a mutant vector using Equation 10:


$$\begin{array}{l} y_{i}=x_{r_{1}}+F_{i}\left(x_{r_{2}}-x_{r_{3}}\right) \end{array}$$ (10)

where *y*_*i*_ is the mutant vector for target *x*_*i*_, the indices *r*_1_, *r*_2_, and *r*_3_ identify mutually distinct population members, and *F*_*i*_ is the differential scale factor. The implementation then applies either coordinate-space or covariance-eigenvector-space crossover.

The IDE-related operator uses a rank- and stage-dependent reference, as shown in Equation 11:


$$\begin{array}{l} y_{i}=b_{i}+F_{o}\left(x_{r_{1}}-x_{o}\right)+F_{o}\left(x_{r_{2}}-{\tilde{x}}_{r_{3}}\right) \end{array}$$ (11)

Here, *x*_*o*_ and *F*_*o*_ depend on the target's rank and the current search stage; *b*_*i*_ is selected from *x*_*o*_ or *x*_*i*_; and selected coordinates of ${\tilde{x}}_{r_{3}}$ may be randomized within their bounds. A tilde identifies the modified reference vector.

The jSO-related current-to-pbest operator is given by Equation 12:


$$\begin{array}{l} y_{i}=x_{i}+F_{w,i}\left(x_{pbest}-x_{i}\right)+F_{i}\left(x_{r_{1}}-{\tilde{x}}_{r_{2}}\right) \end{array}$$ (12)

where *x*_*pbest*_ is sampled from the best fraction of the population, *x*_*r*_1__ is a distinct population member, and ${\tilde{x}}_{r_{2}}$ is sampled from the union of the population and the external archive. The weighted scale factor is defined in Equation 13:


$$F_{w,\;i}=\{\begin{array}{cc} 0.7F_{i}, & \rho <0.2,\\ 0.8F_{i}, & 0.2\leq \rho <0.4,\\ 1.2F_{i}, & \rho \geq 0.4. \end{array}$$ (13)

The CoBiDE-, IDE-, and jSO-related operators retain their own parameter adaptation and crossover procedures; the displayed equations identify their principal mutation structures.

#### 2.3.3 Feedback-based operator selection and stage policy

Let *n*_*h*_ be the feedback counter for operator *h*∈{1, 2, 3, 4}. The index mapping is *h* = 1 for CoBiDE, *h* = 2 for IDE, *h* = 3 for the SAO-derived operator, and *h* = 4 for jSO. All four counters are initialized to 2. Roulette selection uses the probabilities in Equation 14:


$$\begin{array}{l} p_{h}=\frac{n_{h}}{\sum_{\ell =}^{4}n_{\ell }} \end{array}$$ (14)

where *p*_*h*_ is the probability of selecting operator *h*, and ℓ is the dummy index over the four operators. After greedy replacement, the counter $n_{\tilde{h}}$ associated with the executed operator is increased by the number of accepted trials for the CoBiDE-, IDE-, and SAO-derived operators, but only by the number of strictly improving trials for the jSO-derived operator. Equal-cost trials may replace their parents in all four branches, but they do not increase the jSO feedback counter. These counters are distinct from the escape-event counts in Table 2. Immediately after an operator is sampled, if $\underset{h}{\min}p_{h}<0.05$, all counters are reset to 2 to remove the accumulated imbalance. Feedback from the current generation is added before the next draw, so equal probabilities at that draw are not guaranteed. The reset does not change the operator already sampled. Actual execution remains subject to the stage-dependent remapping below.

**Table 2:** Reported stagnation-escape diagnostics over 30 runs.

Method	Mean trapping events per run	Successful escapes across 30 runs	Event-based escape rate
DCFO	6.0	28	15.56%
DE	7.5	15	6.67%
CoBiDE	6.8	11	5.39%
jSO	7.2	13	6.02%

The mean trapping count is averaged over 30 runs; successful escapes are counted cumulatively across those runs. The escape percentage equals 100 times the cumulative escape count divided by the product of the reported mean trapping count and 30. For the DCFO, 28 escapes divided by the product of 6.0 and 30 yields 15.56%. This is an event-based rate, not the percentage of runs that succeed.

After an operator *h* is sampled, the implementation applies the stage-dependent remapping in Equation 15 before execution:


$$\tilde{h}=\{\begin{array}{cc} 4, & 0.2<\rho \leq 0.4\;and\;h=3,\\ 2, & \rho >0.4\;and\;h\in \{3,4\},\\ h, & otherwise. \end{array}$$ (15)

Thus,$\;\tilde{h}$ is the executed operator index. The rule redirects the SAO-derived operator to jSO during the middle stage and redirects both of those selections to IDE after 40% of the nominal budget. It does not transfer feedback counts between operators. At the first crossing of ρ = 0.2, the algorithm also replaces the current population once with uniformly sampled candidates.

The population size is reduced from its initial value *N*_0_ toward *N*_*min*_ = 10 according to Equation 16:


$$\begin{array}{l} N\left(\rho \right)=\text{round}\left[N_{0}+\left(N_{min}-N_{0}\right)\rho \right] \end{array}$$ (16)

Here, round(·) denotes rounding to the nearest integer. When the target size decreases, the worst candidates are removed, and the archive is truncated to remain compatible with the active population.

Figure 2 compares three operator-selection schemes. The DCFO combines historical operator feedback weighting with the stage-dependent remapping described above; model A selects operators with equal probability, and model B uses historical operator feedback weighting without stage-dependent remapping. This comparison examines selection behavior within the optimizer; it does not isolate all components of the DCFO or constitute a robot-planning comparison.

**Figure 2:**
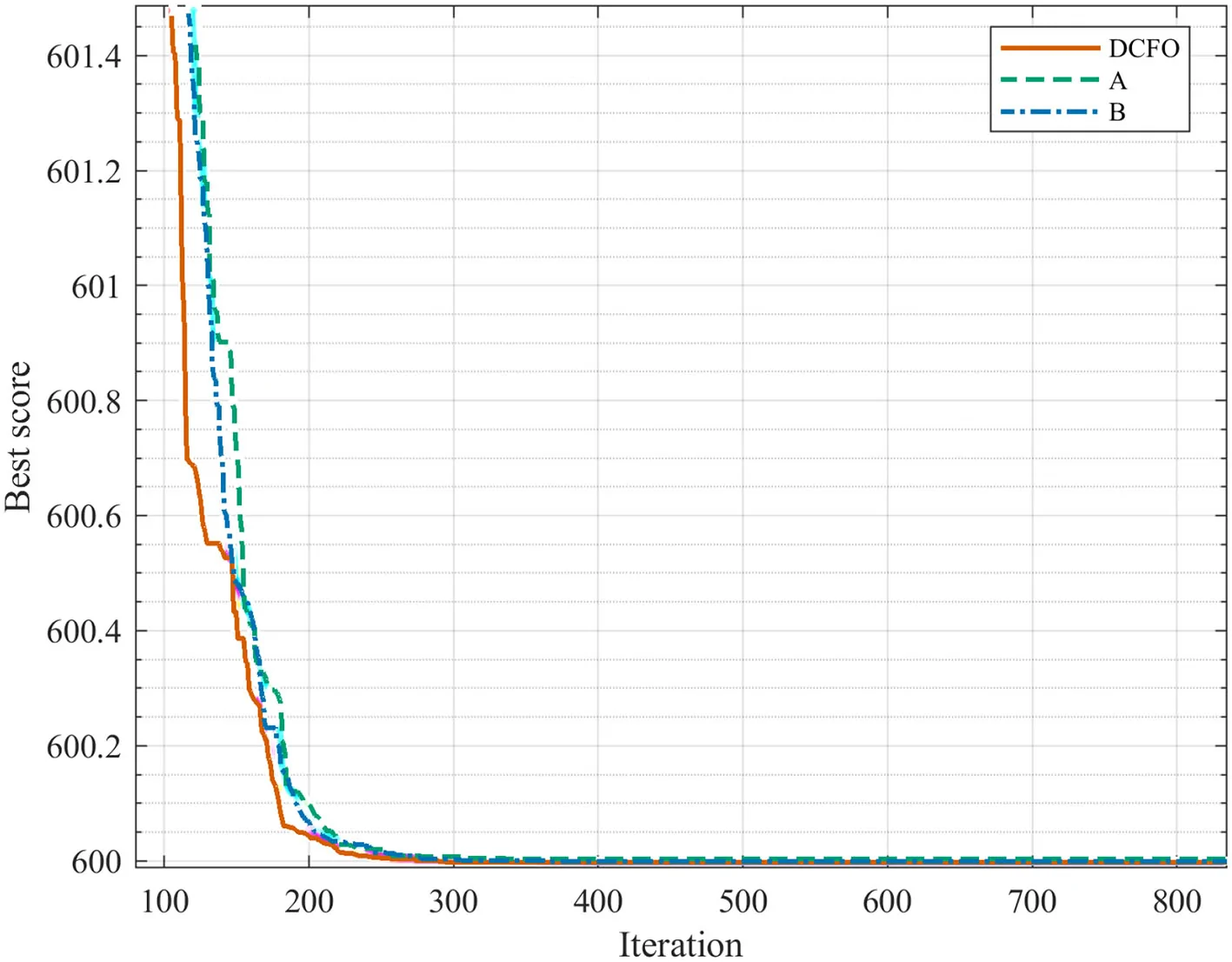
Convergence comparison of three operator selection schemes. The DCFO (orange solid line) combines historical operator feedback weighting with stage-dependent operator remapping. Model A (green dashed line) uses equal-probability random selection. Model B (blue dash-dot line) uses historical operator feedback weighting without stage-dependent remapping. The ordinate is the best objective value, and the abscissa is the iteration number. A and B denote model variants, not figure panels.

#### 2.3.4 Stagnation-triggered covariance perturbation

When all current fitness values are identical, every candidate is perturbed before operator execution. Let the retained history contain *n* solution vectors *x*^(*q*)^ with mean $\bar{x}$. The sample covariance is given by Equation 17:


$$\begin{array}{l} \Sigma_{t}=\frac{1}{n-1}\overset{n}{\underset{q=1}{\sum }}\left(x^{\left(q\right)}-\bar{x}\right){\left(x^{\left(q\right)}-\bar{x}\right)}^{T} \end{array}$$ (17)

Here, *q* indexes the retained history vectors and Σ_*t*_ is their sample covariance at search state *t*. The history contains at most 100 vectors; if fewer than two are available, the implementation uses the identity matrix. The covariance is symmetric and positive semidefinite. With consistent left and right singular vectors in the decomposition $\Sigma_{t}=Udiag\left(s_{1},\ldots ,s_{D}\right)V^{T}$, the regularized inverse is given by Equation 18:


$$\begin{array}{l} {\left(\Sigma_{t}+\lambda I_{D}\right)}^{-1}=Udiag\left(\frac{1}{s_{1}+\lambda },\ldots ,\frac{1}{s_{D}+\lambda }\right)V^{T} \end{array}$$ (18)

where the columns of *U* and *V* are the left and right singular vectors, *s*_*j*_ is the *j*-th singular value, *I*_*D*_ is the *D*×*D* identity matrix, and λ>0 regularizes the inverse. Equations 19 and 20 define the coordinate mask and periodic amplitude, respectively:


$$\begin{array}{l} q_{t}=\frac{t}{t+1},m_{t,j}\sim \text{Bernoulli}\left(q_{t}\right) \end{array}$$ (19)

$$\begin{array}{l} c_{t}=1+\cos\left(\frac{2\pi \text{mod}\left(t,\tau \right)}{\tau }\right) \end{array}$$ (20)

Here, *c*_*t*_ is the periodic amplitude factor and mod(*t*, τ) is the remainder after division of *t* by τ. The variable *t* is the current *FES* value supplied to the perturbation routine, *q*_*t*_ is the probability of perturbing a coordinate, and *m*_*t*_ is a binary mask. With *z*_*t*_~*N*(0, *I*_*D*_), Equations 21 and 22 define the perturbation row vector and masked update, respectively:


$$\begin{array}{l} \Delta_{t}=\sigma c_{t}z_{t}{\left(\Sigma_{t}+\lambda I_{D}\right)}^{-1} \end{array}$$ (21)

$$x\prime_{t,i}=x_{t,i}+m_{t}⊙\Delta_{t}$$ (22)

Here, *x*_*t, i*_ is candidate *i*, ⊙ denotes element-wise multiplication, σ is the perturbation strength, and τ is the modulation period. The parameter values are λ = 0.05, σ = 0.2, and τ = 60.

#### 2.3.5 Workflow and computational complexity

Figure 3 illustrates the behavioral organization of the DCFO. Each replanning call begins with population initialization. Feedback-based sampling selects an operator, and stage-dependent remapping determines which operator executes. Triggered stagnation perturbation and the scheduled one-time population refresh precede trial generation. Only the selected, remapped operator generates trials in each generation, followed by greedy replacement, feedback updates, and population reduction. The optimizer returns a joint command to the simulator, which updates the scene and starts the next replanning call. The arrows in Figure 3 represent behavioral relationships, not sequential execution of all four operators.

**Figure 3:**
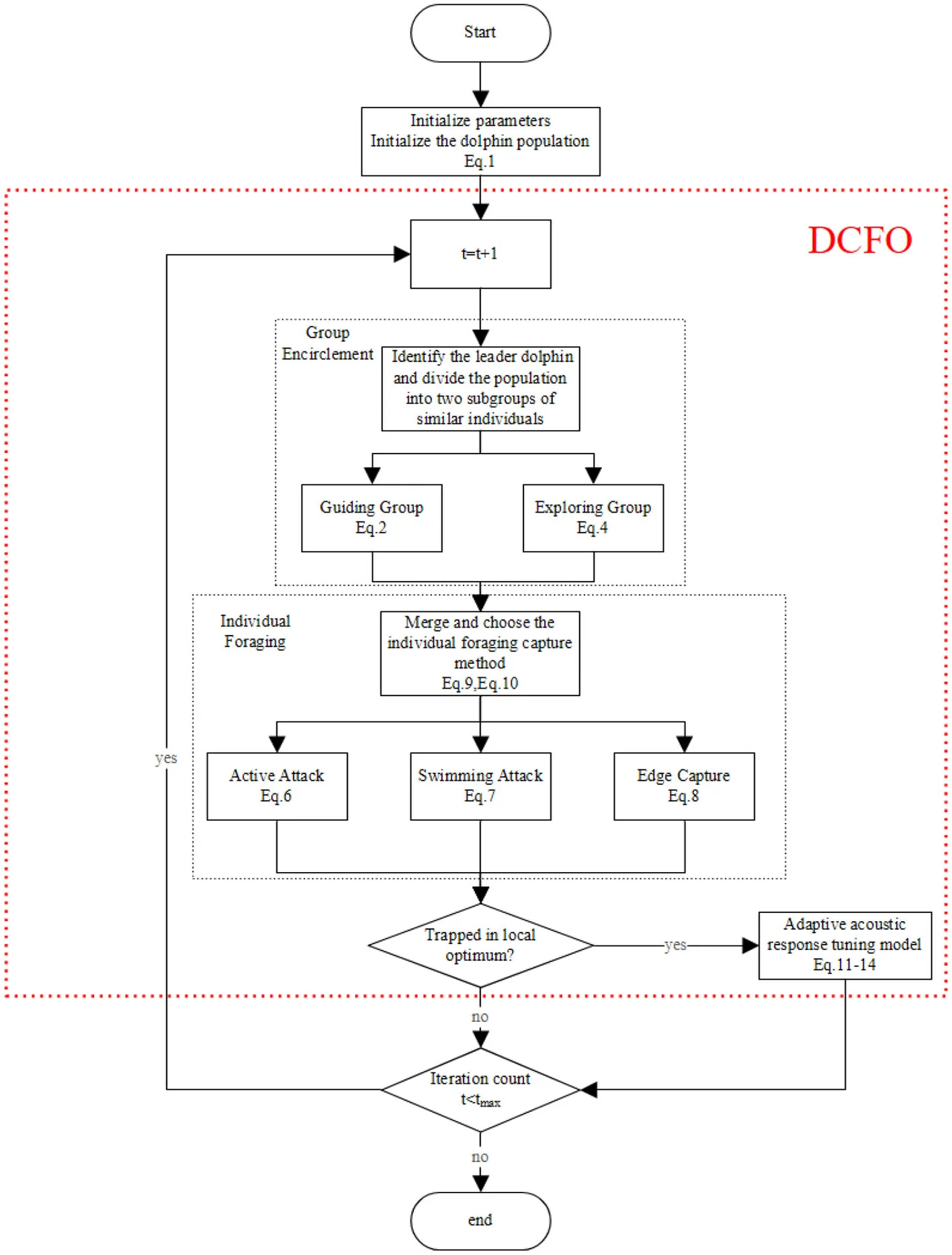
Behavioral organization of the DCFO. Block correspondence with the method description is as follows: initialization, Section 2.3; “Guiding Group” and “Exploring Group,” Section 2.3.1; “Active Attack,” “Swimming Attack,” and “Edge Capture,” the CoBiDE-, IDE-, and jSO-related operators in Section 2.3.2; method selection, Section 2.3.3; and “Adaptive acoustic response tuning model,” the numerical inverse covariance perturbation in Section 2.3.4. The acoustic terminology is a behavioral analogy, not an acoustic sensor or neural model. Equation labels inside the original schematic use its earlier numbering and do not refer to equations in this manuscript. The four operators are alternatives, with only one executed per generation and with the triggered perturbation preceding trial generation. Section 2.3.5 specifies the execution order.

Ignoring fitness evaluation, coordinate updates require approximately *O*(*TND*) operations for *T* generations, population size *N*, and dimension *D*. Covariance construction costs *O*(*HD*^2^) and singular value decomposition costs *O*(*D*^3^) whenever the stagnation perturbation is triggered, where *H* ≤ 100 is the retained-history length. In the reported simulations, *D*∈{12, 24}. Total runtime also depends on the number and cost of objective evaluations, as well as on simulation overhead.

### 2.4 Experimental design

#### 2.4.1 Numerical benchmark protocol

The DCFO was first compared with the Snow Ablation Optimizer (SAO; Deng and Liu, 2023), EA4eig (Bujok and Kolenovsky, 2022), the Gazelle Optimization Algorithm (GOA; Abdel-Salam et al., 2024), the Chernobyl Disaster Optimizer (CDO; Shehadeh, 2023), RIME (Su et al., 2023), and the Subtraction-Average-Based Optimizer (SABO; Trojovsky and Dehghani, 2023) on the CEC2020 and CEC2022 single-objective benchmark suites (Yue et al., 2020; Kumar et al., 2022). The benchmark protocol used a population of 30, a nominal budget of 5,000 function evaluations, and 30 independent runs for each reported function and dimension. Both 10- and 20-dimensional cases were evaluated.

Benchmark performance was evaluated using the best, worst, mean, and standard deviation of the final objective values across 30 independent runs. Mean error was defined as the mean final objective value minus the function-specific optimum. The results are compared descriptively. Detailed benchmark results are provided in Supplementary Tables S1–S6 and Supplementary Figures S1–S3.

The nominal counter FES excludes objective reevaluations during the one-time population refresh and stagnation recovery. Therefore, MFES limits the counted evaluations rather than all objective calls. Both the benchmark and robot comparisons use matched nominal settings; equality of total objective-call counts is not assumed.

#### 2.4.2 Multi-robot simulation protocol

Simulations were implemented in MATLAB R2020a and run on Windows 10 with 16 GB RAM and an Intel Core i5-9300H processor at 2.40 GHz. The workspace measured 100 cm by 100 cm. Scenario 1 contained six robots, seven static obstacles, and three uniformly moving dynamic obstacles. Scenario 2 contained 12 robots, 14 static obstacles, and 6 moving obstacles. Figure 4 shows the initial robot, goal, obstacle, and motion configurations.

**Figure 4:**
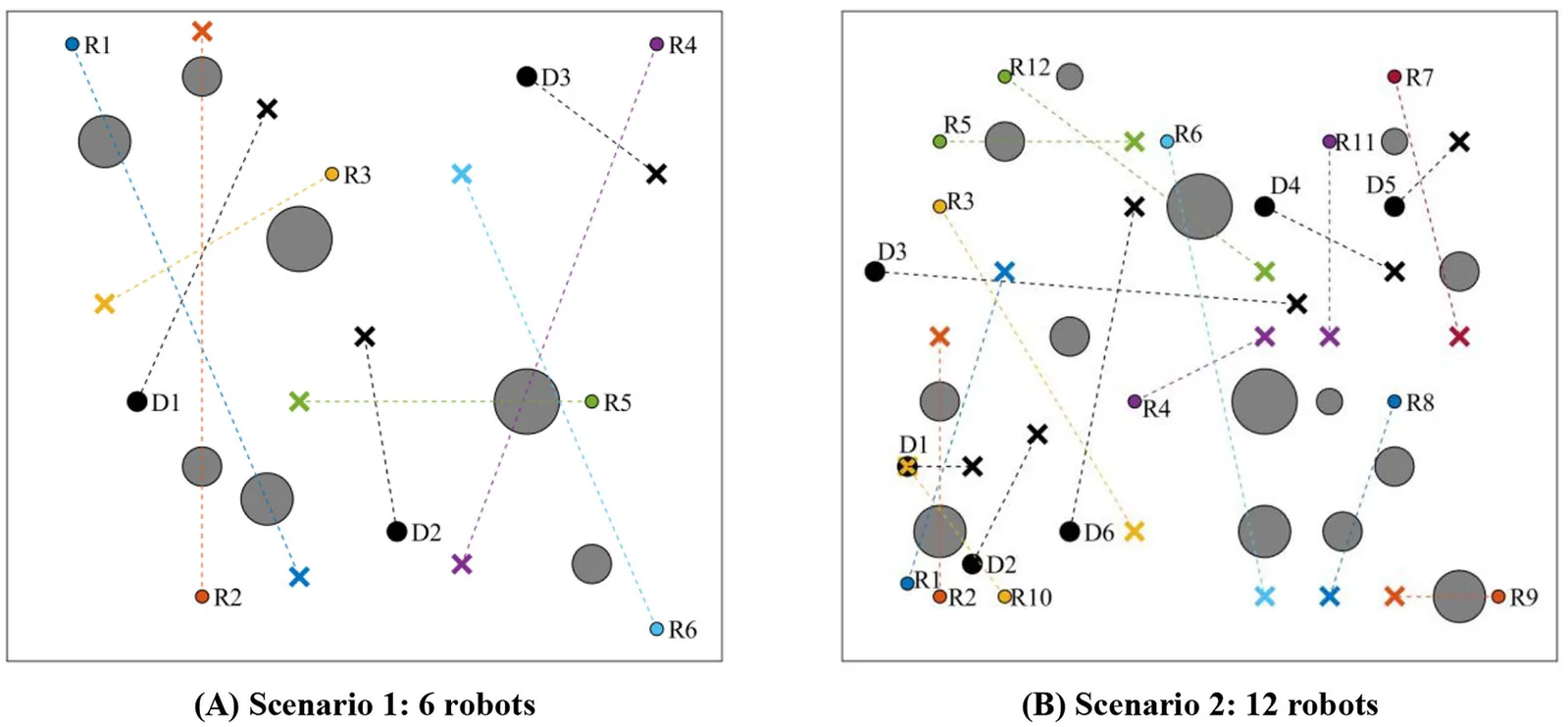
Initial multi-robot planning environments. **(A)** Scenario 1: Six robots, seven static obstacles, and three dynamic obstacles. **(B)** Scenario 2: 12 robots, 14 static obstacles, and six dynamic obstacles. Colored circles mark robot starting positions, colored crosses mark goals, gray circles represent static obstacles, and black circles and crosses indicate dynamic obstacle states and motion directions.

For optimizer-based planners, the initial population was *N* = 30 and each replanning call used a nominal counter limit of *MFES* = 2, 000. The command dimension was *D* = 12 for Scenario 1 and *D* = 24 for Scenario 2. The DCFO was compared with the Superb Fairy-wren Optimization Algorithm (SFOA; Jia et al., 2025), the multi-strategy self-adaptive differential sine–cosine algorithm (sdSCA; Akay and Yildirim, 2023), the SAO, EA4eig, and the Dynamic Window Approach (DWA; Fox et al., 1997). Each method was evaluated over 30 runs in each scenario.

Performance measures included aggregate path length, average execution time (AET), total robot movement steps, and total fitness. Aggregate path length sums the distances traveled by individual robots; total movement steps sum their step counts rather than joint replanning cycles. Total fitness sums optimizer-returned costs over replanning calls. AET measures average simulation runtime, including visualization and pauses, rather than isolated optimizer runtime or control-cycle latency. The non-finite DWA fitness output is excluded from cost comparisons.

## 3 Results

### 3.1 Operator selection and stagnation recovery

In Figure 2, the DCFO approaches the common lower plateau earlier than the two simplified selection models. Table 2 compares its stagnation-escape behavior with that of standard differential evolution (DE), CoBiDE, and jSO. The event-based escape rate is the total number of successful escapes divided by the product of the mean trapping count per run and 30 runs. The DCFO recorded 28 successful escapes and the highest reported escape rate (15.56%). These results describe the escape behavior of the complete algorithms.

### 3.2 Online multi-robot path planning

Table 3 summarizes the scenario-level results. In the six-robot scenario, the DCFO produced an aggregate path length of 448.94 cm. The next shortest result was 461.57 cm for the sdSCA, corresponding to a 2.74% reduction. The sdSCA recorded the lowest AET in this smaller problem, whereas the DCFO provided the shortest path and the lowest finite total fitness.

**Table 3:** Simulation-based online multi-robot path-planning performance.

Scenario	Method	Total robot movement steps	Aggregate path length (cm)	AET (s)	Total fitness
6 robots	SFOA	761	932.06	79.58	31,500
6 robots	sdSCA	381	461.57	40.21	15,300
6 robots	SAO	421	477.06	46.43	17,100
6 robots	EA4eig	405	469.32	60.27	16,000
6 robots	DWA	353	519.50	135.00	Inf
6 robots	DCFO	376	448.94	54.19	14,900
12 robots	SFOA	1484	1833.71	196.00	90,600
12 robots	sdSCA	575	690.73	77.02	14,700
12 robots	SAO	568	632.90	87.00	34,600
12 robots	EA4eig	498	598.54	90.69	13,100
12 robots	DWA	429	625.20	313.55	Inf
12 robots	DCFO	452	548.86	74.75	11,400

Total robot movement steps sum the individual robots' step counts, not joint replanning cycles. Aggregate path length sums the per-robot average distances. AET is average simulation execution time in seconds, including visualization and pauses, rather than isolated optimizer runtime or control-cycle latency. Total fitness sums optimizer-returned costs over replanning calls. Inf denotes the non-finite DWA fitness output and is excluded from cost comparisons; it does not, by itself, indicate navigation failure. Comparisons are descriptive. Detailed records are provided in Supplementary Tables S7–S12.

In the 12-robot scenario, the DCFO produced an aggregate path length of 548.86 cm, compared to 598.54 cm for the next shortest method, EA4eig. The difference corresponds to an 8.30% reduction. The DCFO also recorded the lowest AET (74.75 s) and the lowest finite total fitness (11,400).

The results show a trade-off between path length, movement steps, and runtime. The DWA had the fewest total movement steps in both scenarios, whereas the DCFO had the shortest aggregate path. This difference reflects the variable displacement per step. In Scenario 1, the DCFO produced a shorter path than the sdSCA but had a longer recorded runtime. Figures 5, 6 illustrate representative trajectories and the larger detours produced by several comparators. Detailed robot-level results are provided in Supplementary Tables S7–S12.

**Figure 5:**
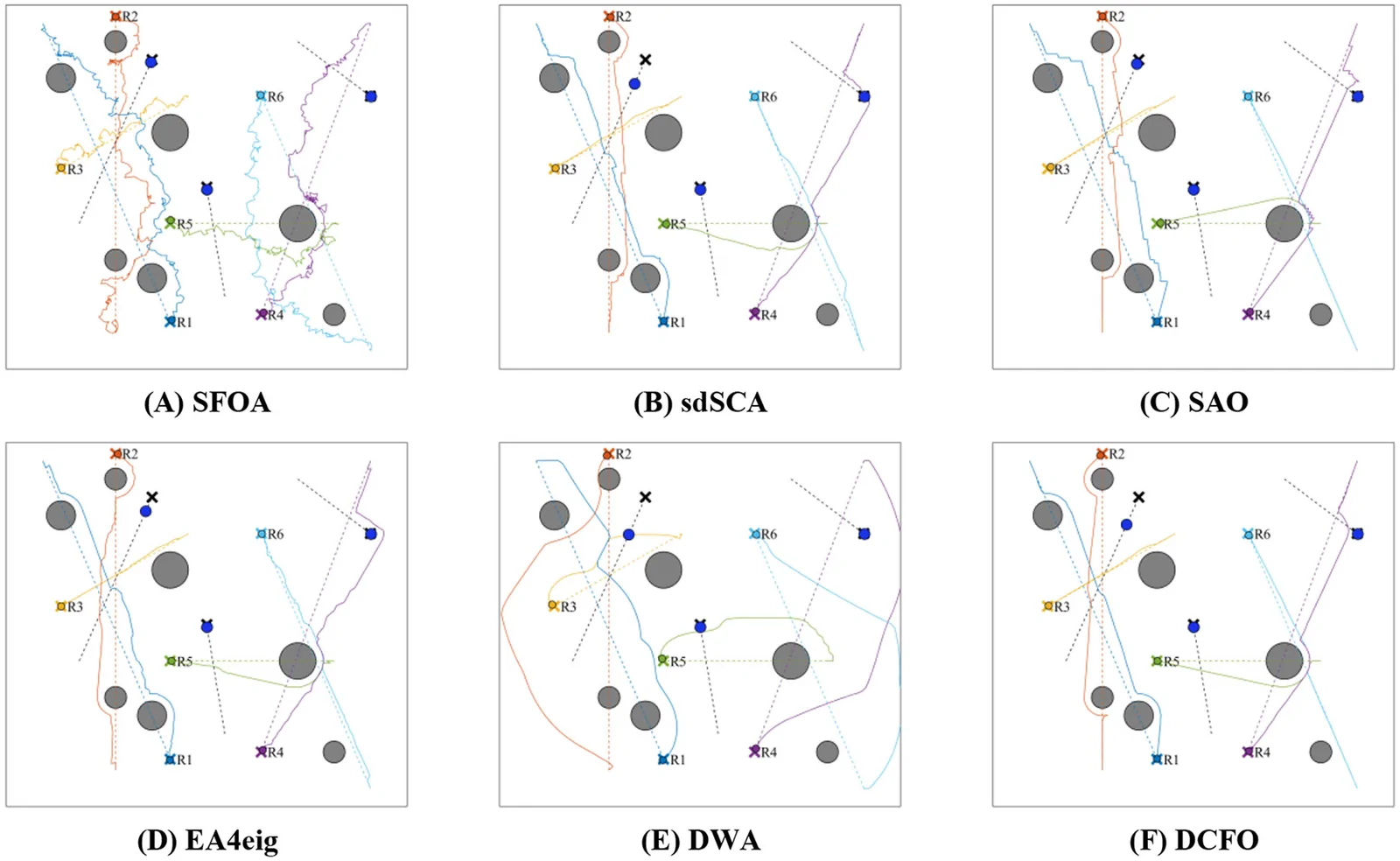
Representative robot trajectories in the six-robot scenario. **(A)** SFOA, **(B)** sdSCA, **(C)** SAO, **(D)** EA4eig, **(E)** DWA, and **(F)** DCFO. Each panel shows one run under the same scenario configuration. Aggregate numerical comparisons are reported in Table 3.

**Figure 6:**
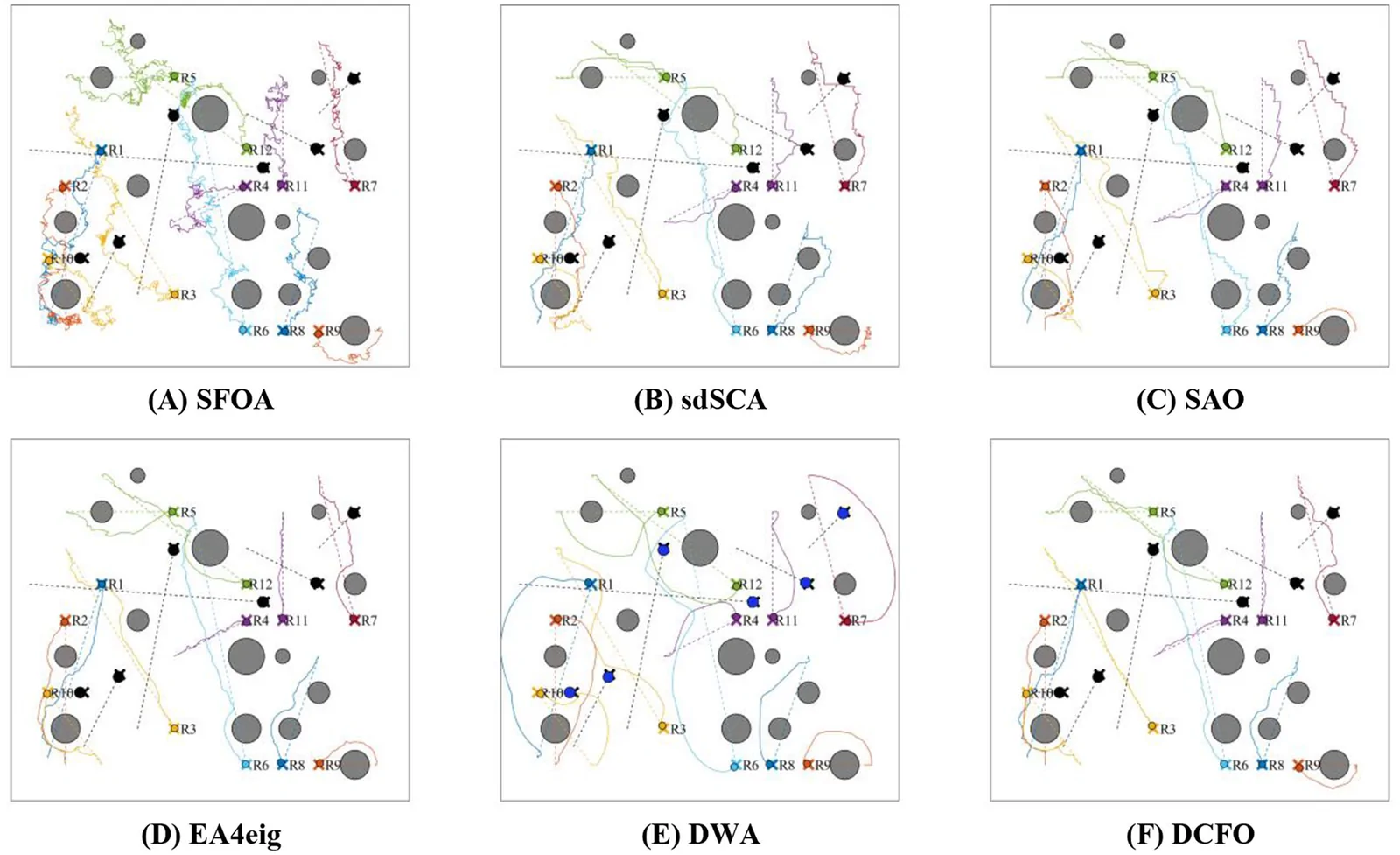
Representative robot trajectories in the 12-robot scenario. **(A)** SFOA, **(B)** sdSCA, **(C)** SAO, **(D)** EA4eig, **(E)** DWA, and **(F)** DCFO. Each panel shows one run under the same scenario configuration. Aggregate numerical comparisons are reported in Table 3.

Figure 7 compares the average path length values of individual robots. The DCFO obtained the shortest average path for five of the six robots in Scenario 1 and eight of the 12 robots in Scenario 2. The SAO produced the shortest path for Robot 3 in Scenario 1. In Scenario 2, EA4eig produced shorter paths for Robots 6, 9, and 11, while the SAO produced the shortest path for Robot 10. The aggregate advantage of the DCFO therefore accompanies shorter paths for several team members, although the best method differs for some individual robots. These counts compare reported robot-level means, not the frequency of successful runs. Detailed values are provided in Supplementary Tables S8, S11.

**Figure 7:**
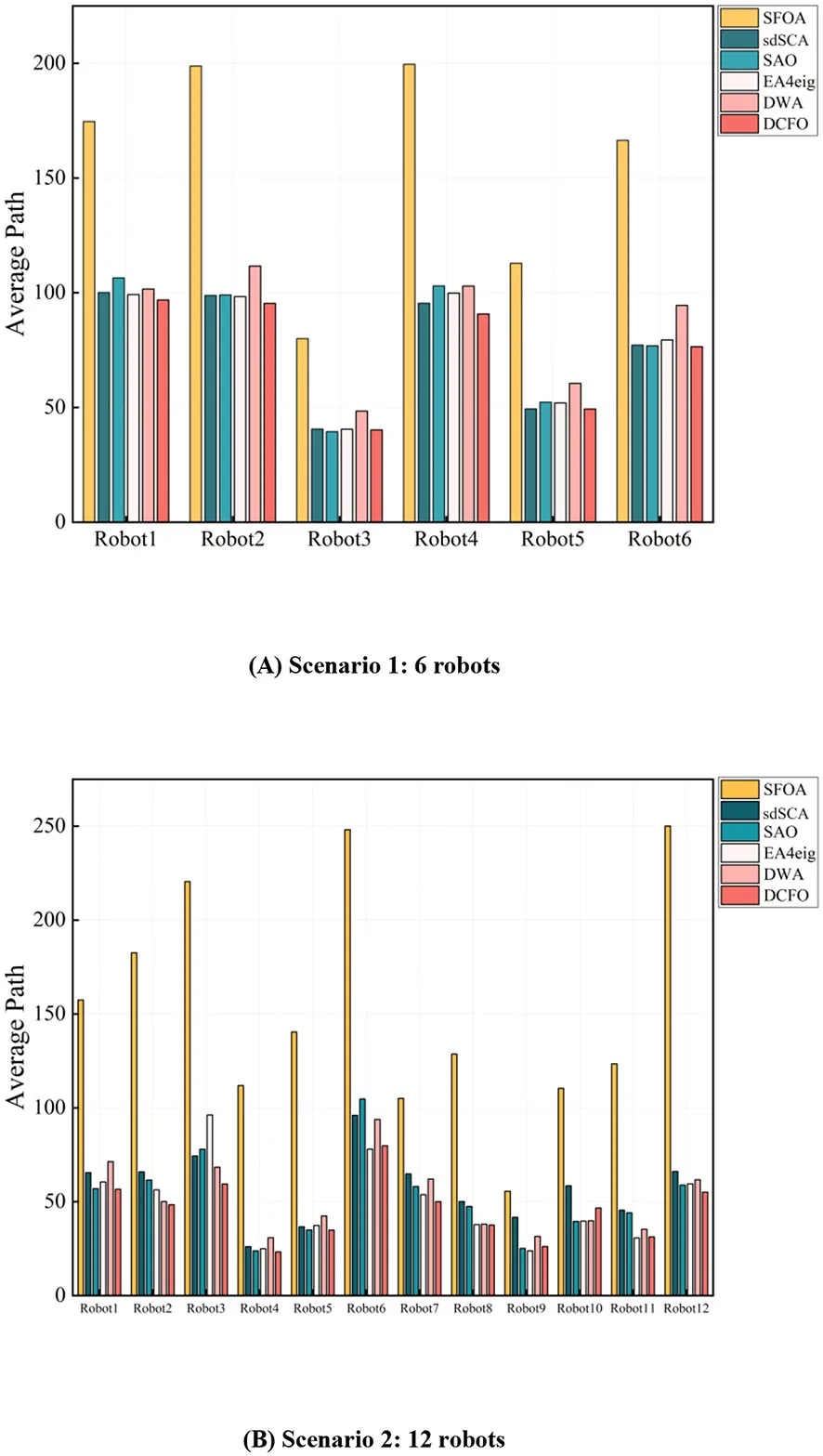
Per-robot average path lengths in the two simulation scenarios. **(A)** Scenario 1: six robots. **(B)** Scenario 2: 12 robots. Each bar represents the average path length of one robot under the indicated method. Path length is measured in centimeters. Summing the robot-level averages provides the aggregate path length reported in Table 3, subject to rounding. Detailed values are provided in Supplementary Tables S8, S11.

Figure 8 plots mean fitness against simulation steps in the outer replanning loop, rather than iterations within a single optimizer call. In Scenario 1, the SAO initially declined rapidly, while the DCFO, sdSCA, and EA4eig approached similar lower displayed levels ahead of the SFOA. In Scenario 2, the DCFO and EA4eig showed similar early trends, after which the DCFO approached the displayed baseline earlier. The curves describe progress through successive planning problems as the scene changes. The DWA is omitted because its recorded fitness is non-finite. Further details are provided in Supplementary Section S2.

**Figure 8:**
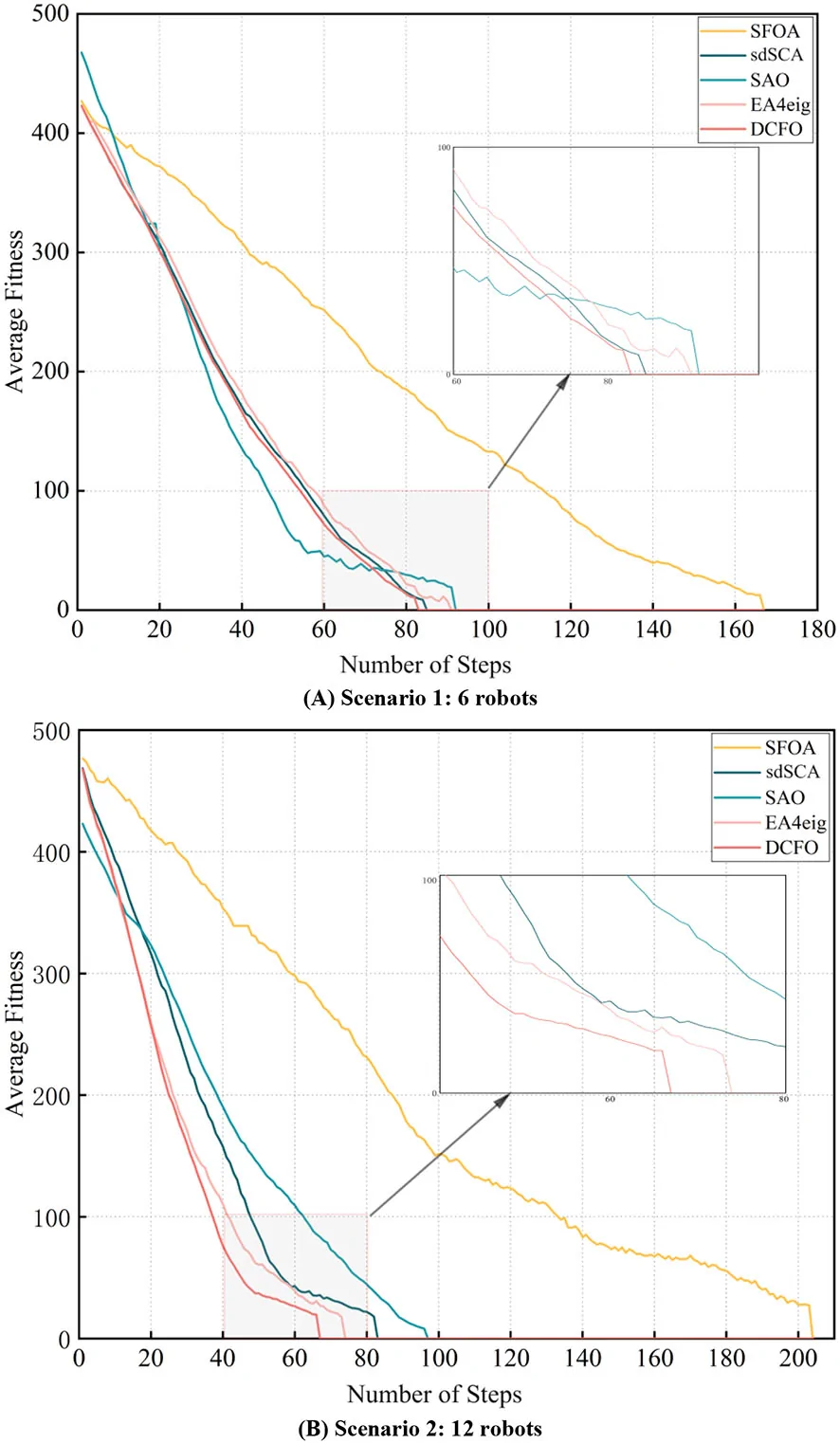
Mean-fitness trajectories during multi-robot simulation. **(A)** Scenario 1: 6 robots. **(B)** Scenario 2: 12 robots. The horizontal axis denotes simulation steps in the outer replanning loop, not iterations within one optimizer call. The planning objective changes with the scene, unlike the fixed benchmark functions in Figure 9. Insets enlarge the terminal region. The DWA is omitted because its fitness is non-finite. Further details are provided in Supplementary Section S2.

### 3.3 Numerical benchmark performance

The benchmark comparisons use the nominal settings in Section 2.4.1; the DCFO refresh and recovery evaluations are outside the stopping counter, so actual objective-call counts are not assumed to be equal across methods. At the reported precision, the DCFO attained or tied the lowest mean error on 7 of 10 CEC2020 functions at *D* = 10 and on 9 of 12 CEC2022 functions at each dimension. These counts include ties. Detailed function-level results are provided in Supplementary Tables S1–S3.

Table 4 presents the mean final objective errors for the six 10-dimensional functions shown in Figure 9. The results distinguish performance across the selected functions. For CEC2020 F3, EA4eig achieved a slightly lower mean error than the DCFO (26.9257 vs. 27.1249). The DCFO obtained lower errors on F6 (1.8532 vs. 5.4993) and F9 (419.8193 vs. 421.5758). For CEC2022, the DCFO and EA4eig both reached zero mean error at the reported precision on F3. The DCFO obtained lower mean errors on F6 (0.3430 vs. 0.6743) and F11 (5.0142 vs. 56.6667). Thus, the difference between these two leading methods ranges from a tie or a small difference to a larger separation, depending on the function.

**Figure 9:**
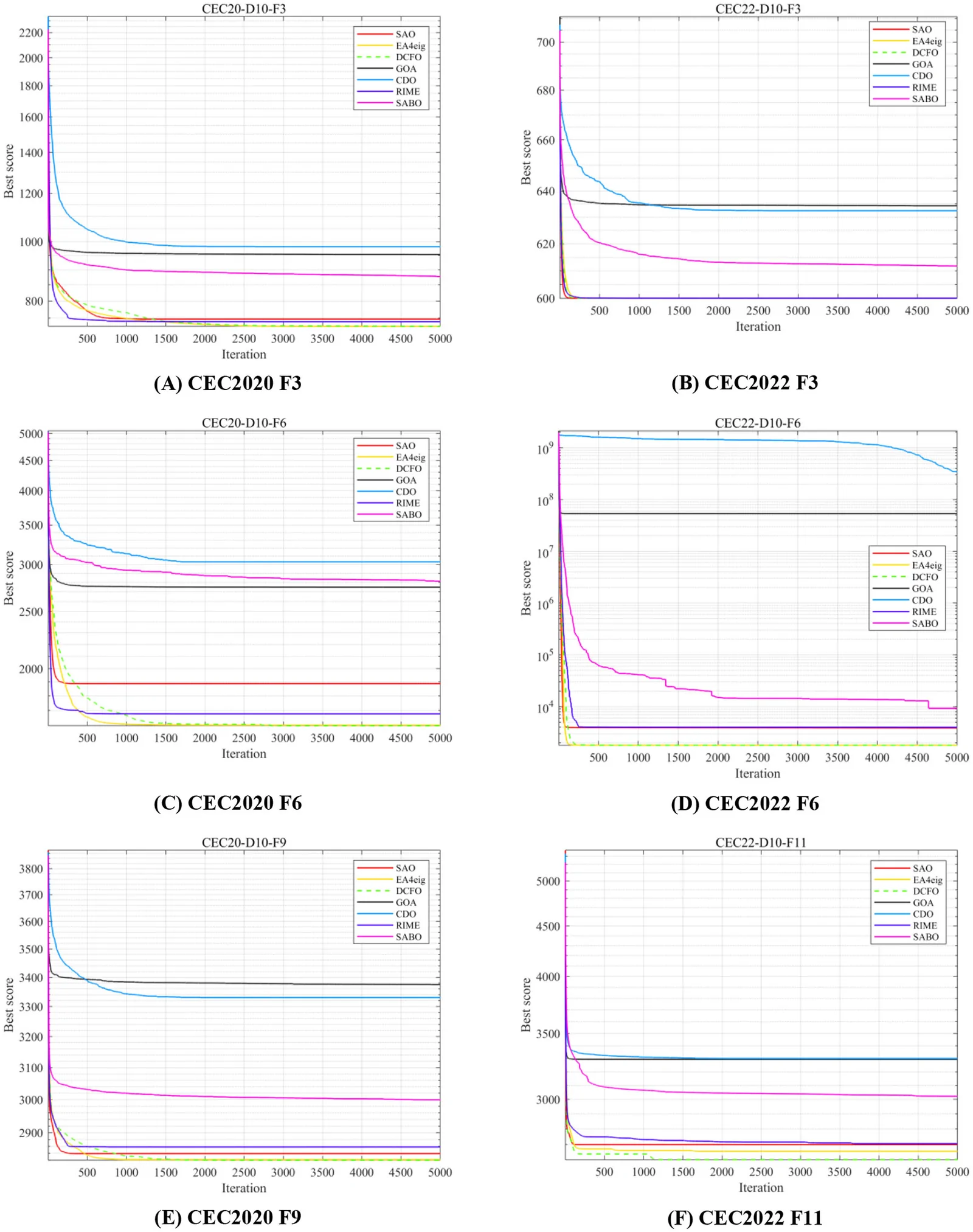
Representative convergence curves on 10-dimensional benchmark functions. CEC2020 functions appear in the left column: **(A)** F3, **(C)** F6, and **(E)** F9. CEC2022 functions appear in the right column: **(B)** F3, **(D)** F6, and **(F)** F11. Additional 10- and 20-dimensional panels are provided in Supplementary Figure S1 and Supplementary Section S1.

**Table 4:** Mean final objective errors on the six 10-dimensional benchmark functions shown in Figure 9.

Benchmark	SAO	EA4eig	GOA	CDO	RIME	SABO	DCFO
CEC2020 F3	47.11	**26.926**	253.21	281.82	39.723	176.9	27.125
CEC2020 F6	287.54	5.4993	1147.7	1432.6	77.713	1212	**1.8532**
CEC2020 F9	438.99	421.58	974.83	930.33	458.02	599.76	**419.82**
CEC2022 F3	0.003643	**0**	34.326	32.434	0.011498	11.721	**0**
CEC2022 F6	2065.4	0.6743	5.361E + 07	3.47E + 08	2181.2	7478.3	**0.34304**
CEC2022 F11	98.563	56.667	694.68	700.94	104.6	421.87	**5.0142**

Mean error is the mean final objective value over 30 runs minus the function-specific optimum. Values are shown to at most five significant digits; bold indicates the lowest value in each row, including ties. Comparisons use matched nominal settings; refresh and recovery evaluations are excluded from the DCFO stopping counter, so equal total objective-call counts are not assumed. The functions correspond to Figure 9. Complete benchmark results are provided in Supplementary Tables S2, S3.

The convergence profiles in Figure 9 complement these final-error comparisons. On CEC2020 F3, F6, and F9, the DCFO and EA4eig finished near the lower objective levels, whereas several other methods settled at higher plateaus. The CEC2022 F3 curves approach a common lower level for several methods, while the F6 and F11 panels show more distinct final levels. This variation is consistent with the function-dependent mean errors in Table 4. The curves show best objective values during search, whereas Table 4 reports final mean errors relative to the function-specific optima over 30 runs. Additional convergence curves and final-value distributions are provided in Supplementary Figures S1, S2.

The descriptive average of the four Friedman summary ranks was 1.72 for the DCFO, followed by 1.89 for EA4eig. This average is the arithmetic mean of the ranks for the best, worst, standard deviation, and mean final objective values, rather than a pooled Friedman analysis. The DCFO had the lowest average among the compared methods, consistent with its competitive function-value summaries. Detailed rank results are provided in Supplementary Table S6 and Supplementary Figure S3.

## 4 Discussion

### 4.1 Interpretation and performance trade-offs

The DCFO achieved shorter aggregate paths in both tested robot scenarios and the lowest recorded simulation runtime in the 12-robot scenario. In the six-robot scenario, the sdSCA was faster, illustrating the trade-off between path quality and computation. The benchmark comparisons complement these navigation results by characterizing the search behavior of the operator portfolio across different objective landscapes. To gether, the results support further development of feedback-based operator coordination for repeated motion planning.

The robot-level results in Figure 7 help explain the team-level comparison. Shorter aggregate paths are accompanied by improvements for multiple robots, but some robots travel farther under the DCFO than under another method. The planner evaluates joint commands using travel, goal distance, and clearance terms, so its local decisions address the team configuration rather than minimizing each robot's final path independently. Reporting both scales makes this distinction visible. Similarly, the smaller movement-step totals of the DWA do not imply shorter paths because step displacement varies. These measures describe different aspects of navigation and should be considered together when comparing the tested planners.

The portfolio combines elite-guided search with covariance-aware, rank-dependent, and current-to-pbest differential variation strategies. Historical feedback adjusts sampling probabilities, while the stage policy determines which sampled operator is executed. CoBiDE-, IDE-, and SAO-derived operators count accepted trials, including equal-cost replacements; the jSO-derived operator counts strict improvements. These operator-specific feedback rules provide the basis for adaptive selection, distinct from the escape-event measure in Table 2.

The stagnation response uses the geometry of recent solutions to transform a masked Gaussian perturbation through regularized inverse covariance. The cosine factor modulates its amplitude periodically, and identical population fitness values trigger the update. The higher event-based escape rate in Table 2 provides a complementary observation of the complete DCFO algorithm. Component-level studies can further distinguish the contributions of perturbation, population refresh, population reduction, and operator selection.

### 4.2 Applicability and future research

The DCFO translates cooperative foraging into a numerical framework for adaptive joint motion command generation. Its biological inspiration operates at the level of coordinated search behavior. Integrating this optimization-based planner with learned perception or neural control is a potential direction for connecting the framework with brain-inspired robotics (Mompó Alepuz et al., 2024).

The present evaluation covers two dynamic simulation scenarios with ideal state information and a one-step penalty-based objective. Extending the tests to additional team sizes and independently varied obstacle densities would clarify how planning performance changes with task complexity. Physical deployment will also require evaluation under sensing and localization uncertainty, actuator dynamics, and communication delays. Hardware-in-the-loop and robot experiments can assess control-cycle timing and collision avoidance under these conditions.

Further computational evaluation will focus on component contributions, complete objective-call accounting, and planner runtime measured separately from simulation overhead. Retaining individual-run outputs and random seeds will support uncertainty analysis and reproducible statistical comparisons. Extending the framework to three-dimensional motion and multi-objective planning would allow path length, energy use, and inter-robot coordination to be considered within a broader range of tasks.

## 5 Conclusion

This study presents the DCFO, a dolphin-inspired adaptive optimizer for online multi-robot path planning. The method integrates an elite-guided operator and three differential evolution operators with feedback-based selection, stage-dependent remapping, population reduction, and stagnation-triggered inverse covariance perturbation. Benchmark comparisons showed competitive solution quality on the tested CEC2020 and CEC2022 functions. In the dynamic robot simulations, the DCFO achieved the shortest aggregate path in both scenarios, with reductions of 2.74% for six robots and 8.30% for 12 robots relative to the next-best comparator. It also recorded the lowest average simulation execution time in the 12-robot scenario.

Future research will extend DCFO to physical robot platforms and more diverse dynamic environments. Priorities include component-level evaluation, end-to-end timing measurements, and robustness to sensing and communication uncertainty. Three-dimensional and multi-objective planning offer further directions for developing adaptive cooperative navigation.

## Data Availability

The publicly available CEC2020 and CEC2022 benchmark resources used in this study are available at https://github.com/P-N-Suganthan/2020-Bound-Constrained-Opt-Benchmark and https://github.com/P-N-Suganthan/2022-SO-BO, respectively. The aggregate and function-level benchmark data and robot-simulation results supporting the conclusions are included in the article and its Supplementary material.
