# Supplementary material for: Dolphin-inspired adaptive coordination for online multi-robot path planning in dynamic environments
Source: Front Neurorobot. 2026 Oct 29;20:1993109. doi: 10.3389/fnbot.2026.1993109 (PMC13755313; doi:10.3389/fnbot.2026.1993109)
Supplement: Supplementary file 1 [file Supplementary_File_1.docx]

Supplementary Material

**Dolphin-Inspired Adaptive Coordination for Online Multi-Robot Path Planning in Dynamic Environments**

Meijun Liu, Haibin Wu, Lei Qin, Zetong Lu, Rui Cheng and Lijuan Chen

Tables S1-S6 present numerical benchmark results and statistical summaries. Tables S7-S12 report robot movement steps, path lengths, simulation execution times, and total fitness. Figures S1-S3 provide the corresponding graphical results.

# S1 Numerical benchmark results

Tables S1-S3 present the CEC2020 and CEC2022 results. Function labels follow the order used in the experiments: F1-F10 for CEC2020 and F1-F12 for CEC2022. Table S1 reports final objective-value summaries; Tables S2 and S3 report mean errors, with exceptions identified in the table notes. Comparisons use the nominal settings in Section 2.4.1. DCFO refresh and recovery evaluations are excluded from the stopping counter; equality of total objective-call counts is therefore not assumed. Tables S4-S6 contain statistical summaries, and Figures S1-S2 show convergence curves and final-value distributions.

**Supplementary Table S1. Final objective-value summaries for CEC2020 at D = 10.**

| **Function** | **Statistic** | **SAO** | **EA4eig** | **GOA** | **CDO** | **RIME** | **SABO** | **DCFO** |
| --- | --- | --- | --- | --- | --- | --- | --- | --- |
| F1 | best | 102.594 | 100 | 1.21E+10 | 2.09E+10 | 968.9409 | 1.65E+08 | 100 |
| F1 | worst | 10483.53 | 100 | 3.22E+10 | 2.13E+10 | 16019.9 | 5.42E+09 | 100 |
| F1 | std | 2733.612 | 0.00E+00 | 4.94E+09 | 1.1E+08 | 4359.715 | 1.12E+09 | 0.00E+00 |
| F1 | mean | 3280.7 | 100 | 2.11E+10 | 2.12E+10 | 6253.883 | 1.33E+09 | 100 |
| F2 | best | 1740.912 | 1100.28106 | 3375.974 | 3894.564 | 1242.819 | 4805.488 | 1105.1851 |
| F2 | worst | 3707.714 | 1564.44393 | 5350.48 | 5330.462 | 1997.662 | 6159.373 | 1835.17209 |
| F2 | std | 480.062 | 146.084528 | 586.3611 | 347.7628 | 213.5027 | 331.4681 | 172.486318 |
| F2 | mean | 2560.274 | 1273.72299 | 4358.247 | 4657.269 | 1603.411 | 5623.069 | 1380.05648 |
| F3 | best | 732.5221 | 722.147986 | 861.2574 | 954.78 | 715.2181 | 816.2158 | 722.890378 |
| F3 | worst | 766.051 | 737.234567 | 1030.919 | 1009.545 | 752.5414 | 979.4452 | 731.864795 |
| F3 | std | 8.757341 | 3.60753165 | 36.21813 | 13.80909 | 7.285888 | 31.62958 | 2.23309865 |
| F3 | mean | 747.1102 | 726.925661 | 953.208 | 981.8204 | 739.7228 | 876.8984 | 727.124889 |
| F4 | best | 1901.09 | 1900.6709 | 19003.93 | 132463.6 | 1901.549 | 1914.395 | 1900.70714 |
| F4 | worst | 1903.16 | 1902.04127 | 851546.7 | 389208.5 | 1906.22 | 9418.759 | 1902.27861 |
| F4 | std | 0.50011 | 0.32558774 | 230403.8 | 52733.05 | 1.119611 | 1569.913 | 0.32928406 |
| F4 | mean | 1901.927 | 1901.29923 | 234799.4 | 373824.9 | 1902.994 | 2957.983 | 1901.2921 |
| F5 | best | 4923.398 | 1724.49544 | 207440.5 | 215088.9 | 22871.47 | 45276.43 | 1745.5324 |
| F5 | worst | 199515.2 | 2913.43359 | 6384826 | 881801.4 | 318562.1 | 1835048 | 2422.16389 |
| F5 | std | 46448.31 | 208.161033 | 1547731 | 153989.5 | 78686 | 436681.6 | 183.283131 |
| F5 | mean | 72383.12 | 1980.21518 | 2398963 | 513005.6 | 105877.5 | 496021.3 | 2047.99775 |
| F6 | best | 1622.927 | 1600.33588 | 2202.517 | 2763.204 | 1602.022 | 2302.563 | 1600.44096 |
| F6 | worst | 2325.381 | 1720.60587 | 3410.297 | 3669.396 | 1967.144 | 3358.53 | 1607.01289 |
| F6 | std | 182.9455 | 21.7643886 | 307.739 | 171.2671 | 98.27736 | 258.2381 | 1.44813498 |
| F6 | mean | 1887.537 | 1605.4993 | 2747.655 | 3032.618 | 1677.713 | 2812.019 | 1601.85317 |
| F7 | best | 2985.349 | 2104.26019 | 25682.59 | 415275.2 | 4461.064 | 12736.81 | 2100.81934 |
| F7 | worst | 110785.5 | 2390.94536 | 11824995 | 1.58E+08 | 219913.5 | 1472412 | 2385.84192 |
| F7 | std | 29661.31 | 78.1722684 | 2230437 | 56679282 | 66256.09 | 318757.7 | 80.3396744 |
| F7 | mean | 42311.31 | 2243.69554 | 1329988 | 34196645 | 84742.71 | 275626 | 2219.6617 |
| F8 | best | 2300 | 2300 | 4114.051 | 4193.975 | 2300.061 | 2337.15 | 2300 |
| F8 | worst | 4791.272 | 2301.70468 | 6232.085 | 7306.643 | 4932.429 | 3187.163 | 2300 |
| F8 | std | 694.9492 | 0.54176033 | 608.9783 | 1328.259 | 837.0379 | 172.4242 | 2.12E-12 |
| F8 | mean | 2562.955 | 2300.26284 | 5173.092 | 5787.955 | 2820.244 | 2501.487 | 2300 |
| F9 | best | 2813.905 | 2802.9578 | 3156.904 | 3304.926 | 2825.315 | 2904.425 | 2802.38677 |
| F9 | worst | 2874.333 | 2840.09912 | 3701.047 | 3375.42 | 2927.092 | 3141.283 | 2848.51713 |
| F9 | std | 12.71988 | 9.86147094 | 121.0646 | 19.52367 | 22.69944 | 56.0776 | 8.56698889 |
| F9 | mean | 2838.987 | 2821.57579 | 3374.825 | 3330.325 | 2858.021 | 2999.756 | 2819.81933 |
| F10 | best | 2910.252 | 2906.36607 | 3585.574 | 4757.316 | 2910.328 | 2975.822 | 2899.0767 |
| F10 | worst | 3003.26 | 2968.73229 | 5918.556 | 5930.516 | 3014.745 | 3208.231 | 2962.22497 |
| F10 | std | 28.28828 | 21.7754016 | 528.9865 | 214.6655 | 34.66855 | 56.23576 | 18.7231416 |
| F10 | mean | 2937.071 | 2917.46135 | 4351.063 | 5873.714 | 2942.498 | 3063.911 | 2913.88076 |

Note. Best, worst, and mean are summaries of final objective values, not errors relative to the optimum; std denotes the standard deviation of those values. Mean errors in Table S2 subtract the function-specific reference optimum. For F10, the reference value is 2500 (Yue et al., 2020, cited in the main manuscript).

**Supplementary Table S2. CEC2020 benchmark summaries.**

| **Function** | **D** | **SAO** | **EA4eig** | **GOA** | **CDO** | **RIME** | **SABO** | **DCFO** |
| --- | --- | --- | --- | --- | --- | --- | --- | --- |
| F1 | 10 | 3180.7 | 0 | 2.11E+10 | 2.12E+10 | 6153.883 | 1.33E+09 | 0 |
| F2 | 10 | 1460.274 | 173.723 | 3258.247 | 3557.269 | 503.4107 | 4523.069 | 280.0565 |
| F3 | 10 | 47.11019 | 26.92566 | 253.208 | 281.8204 | 39.72285 | 176.8984 | 27.12489 |
| F4 | 10 | 1.927453 | 1.299233 | 232899.4 | 371924.9 | 2.994055 | 1057.983 | 1.2921 |
| F5 | 10 | 70683.12 | 280.2152 | 2397263 | 511305.6 | 104177.5 | 494321.3 | 347.9977 |
| F6 | 10 | 287.537 | 5.4993 | 1147.655 | 1432.618 | 77.71308 | 1212.019 | 1.853173 |
| F7 | 10 | 40211.31 | 143.6955 | 1327888 | 34194545 | 82642.71 | 273526 | 119.6617 |
| F8 | 10 | 362.9552 | 100.2628 | 2973.092 | 3587.955 | 620.2437 | 301.4866 | 100 |
| F9 | 10 | 438.9874 | 421.5758 | 974.8251 | 930.3254 | 458.0206 | 599.7563 | 419.8193 |
| F10 | 10 | 437.0706 | 417.4613 | 1851.063 | 3373.714 | 442.4985 | 563.9112 | 413.8808 |
| F1* | 20 | Inf | 0 | Inf | 0 | 0 | Inf | 0 |
| F2* | 20 | 0 | 0 | Inf | Inf | Inf | Inf | 0 |
| F3 | 20 | 1.178122 | 9.343224 | 0.003961 | 12.1445 | 9.676914 | 1.539871 | 6.340045 |
| F4 | 20 | 0 | 0 | 0 | 0 | 0 | 0 | 0 |
| F5 | 20 | 2321.612 | 20.99151 | 397441.5 | 10176.56 | 4510.413 | 14207.06 | 7.50767 |
| F6 | 20 | 105.2801 | 4.462273 | 388.0774 | 272.8401 | 105.155 | 297.5092 | 0.379569 |
| F7 | 20 | 4134.874 | 0.402527 | 725966.8 | 200447.2 | 155.9959 | 5051.067 | 0.356544 |
| F8 | 20 | 95.68508 | 100.3917 | 512.9069 | 1020.018 | 108.961 | 120.28 | 93.47964 |
| F9 | 20 | 346.5748 | 296.1704 | 428.3331 | 509.775 | 302.2378 | 371.3218 | 287.5771 |
| F10 | 20 | 434.1982 | 421.3725 | 700.2867 | 1082.049 | 423.8536 | 450.1064 | 420.87 |

Note. Mean error is the mean final objective value minus the function-specific optimum. For F10 at D = 10, the optimum is 2500, giving a DCFO mean error of 413.8808. *F1 and F2 at D = 20 are archival summary values with unverified normalization relative to the optimum and are excluded from mean-error comparisons. Inf denotes a nonfinite value.

**Supplementary Table S3. Final mean error on the CEC2022 test-function set.**

| **Function** | **D** | **SAO** | **EA4eig** | **GOA** | **CDO** | **RIME** | **SABO** | **DCFO** |
| --- | --- | --- | --- | --- | --- | --- | --- | --- |
| F1 | 10 | 0 | 0 | 8041.208 | 16254.05 | 0.001008 | 2640.759 | 0 |
| F2 | 10 | 5.16894 | 1.99329 | 606.8685 | 435.1016 | 11.776 | 35.51777 | 0.797316 |
| F3 | 10 | 0.003643 | 0 | 34.3257 | 32.43351 | 0.011498 | 11.72073 | 0 |
| F4 | 10 | 12.53656 | 4.576811 | 33.99407 | 42.71156 | 19.70017 | 37.56114 | 6.036083 |
| F5 | 10 | 0.135292 | 0 | 294.1063 | 422.3707 | 0.128399 | 44.52294 | 0 |
| F6 | 10 | 2065.412 | 0.674298 | 53609996 | 3.47E+08 | 2181.162 | 7478.341 | 0.34304 |
| F7 | 10 | 20.73428 | 0.033165 | 74.76272 | 135.7759 | 12.26689 | 65.67064 | 0.120299 |
| F8 | 10 | 18.69586 | 1.582393 | 47.78525 | 27.40965 | 15.73206 | 66.28575 | 1.593104 |
| F9 | 10 | 229.2844 | 185.5017 | 409.6167 | 356.0564 | 229.2844 | 280.8338 | 185.5017 |
| F10 | 10 | 145.5258 | 118.1807 | 202.9823 | 384.5445 | 136.5262 | 217.7757 | 100.2515 |
| F11 | 10 | 98.56286 | 56.66667 | 694.6799 | 700.9402 | 104.6004 | 421.8694 | 5.014239 |
| F12 | 10 | 165.192 | 146.4726 | 336.3985 | 370.9244 | 164.6043 | 169.4066 | 145.8504 |
| F1 | 20 | 13078.11 | 0 | 29104.64 | 23035.15 | 0.100783 | 18411.83 | 0 |
| F2 | 20 | 38.45737 | 10.19444 | 1294.499 | 1593.624 | 50.51504 | 211.9402 | 12.02068 |
| F3 | 20 | 0.140556 | 4.83E-05 | 61.79908 | 54.70745 | 0.14464 | 38.37484 | 3.24E-06 |
| F4 | 20 | 37.11305 | 25.07294 | 130.4722 | 135.7733 | 52.52107 | 131.6107 | 22.78455 |
| F5 | 20 | 2.034769 | 1.268071 | 1736.854 | 2168.917 | 22.33382 | 815.484 | 0.054386 |
| F6 | 20 | 3344.935 | 134.4902 | 8.77E+08 | 4.53E+09 | 5379.256 | 5798296 | 50.80004 |
| F7 | 20 | 63.2704 | 20.01762 | 166.2466 | 316.4516 | 69.22816 | 166.1088 | 18.94957 |
| F8 | 20 | 41.79972 | 20.97187 | 152.8812 | 43.28518 | 26.97912 | 153.3196 | 20.78157 |
| F9 | 20 | 180.7813 | 165.3441 | 745.7956 | 1171.839 | 180.795 | 327.5745 | 165.4202 |
| F10 | 20 | 762.2526 | 99.16247 | 2468.857 | 3450.406 | 262.852 | 3297.992 | 109.7829 |
| F11 | 20 | 333.3333 | 326.6667 | 4763.318 | 5872.387 | 337.456 | 1856.837 | 286.6667 |
| F12 | 20 | 254.2776 | 200.0044 | 1025.334 | 786.9589 | 250.9317 | 330.5242 | 199.7497 |

Note. Mean error is the mean final objective value minus the function-specific optimum. Comparisons use matched nominal evaluation budgets as defined in Section 2.4.1.

**Supplementary Table S4. Wilcoxon rank-sum summaries for CEC2020 functions.**

| **Function** | **SAO** | **EA4eig** | **GOA** | **CDO** | **RIME** | **SABO** |
| --- | --- | --- | --- | --- | --- | --- |
| F1 | 0 | 0 | 0 | 0 | 0 | 0 |
| F2 | 4.46E-293 | 0 | 0 | 0 | 0.010119 | 0 |
| F3 | 3.00E-226 | 1.13E-236 | 0 | 0 | 8.47E-56 | 0 |
| F4 | 9.44E-42 | 4.29E-13 | 0 | 0 | 4.87E-110 | 0 |
| F5 | 0 | 0 | 0 | 0 | 0 | 0 |
| F6 | 0 | 0 | 0 | 0 | 0 | 0 |
| F7 | 0 | 2.26E-229 | 0 | 0 | 0 | 0 |
| F8 | 0 | 0 | 0 | 0 | 0 | 0 |
| F9 | 0 | 6.16E-292 | 0 | 0 | 0 | 0 |
| F10 | 0 | 0 | 0 | 0 | 0 | 0 |

Note. These archived rank-sum p-values cannot be independently reproduced because the run-level samples, sample sizes, dimensional settings, and test specifications are incomplete. They are not used to establish statistical significance. Zero entries remain unverified.

**Supplementary Table S5. Wilcoxon signed-rank summaries for CEC2020 functions.**

| **Function** | **SAO** | **EA4eig** | **GOA** | **CDO** | **RIME** | **SABO** |
| --- | --- | --- | --- | --- | --- | --- |
| F1 | 0 | 0 | 0 | 0 | 0 | 0 |
| F2 | 2.09E-229 | 0 | 0 | 0 | 2.37E-102 | 0 |
| F3 | 4.59E-101 | 0 | 0 | 0 | 1.16E-09 | 0 |
| F4 | 1.37E-36 | 0 | 0 | 0 | 2.92E-122 | 0 |
| F5 | 0 | 0 | 0 | 0 | 0 | 0 |
| F6 | 0 | 0 | 0 | 0 | 5.59E-166 | 0 |
| F7 | 0 | 3.91E-05 | 0 | 0 | 0 | 0 |
| F8 | 0 | 0 | 0 | 0 | 0 | 0 |
| F9 | 9.29E-278 | 1.40E-07 | 0 | 0 | 0 | 0 |
| F10 | 0 | 0 | 0 | 0 | 0 | 0 |

Note. These archived signed-rank p-values cannot be independently reproduced because the paired run-level samples, sample sizes, dimensional settings, and test specifications are incomplete. They are not used to establish statistical significance. Zero entries remain unverified.

**Supplementary Table S6. Friedman summary ranks.**

| **Method** | **Best-value rank** | **Worst-value rank** | **Std rank** | **Mean-value rank** | **Average rank** |
| --- | --- | --- | --- | --- | --- |
| SAO | 3.4500 | 3.6500 | 3.2396 | 3.4896 | 3.4573 |
| EA4eig | 1.6125 | 2.5625 | 1.7396 | 1.6458 | 1.8901 |
| DCFO | 1.4625 | 2.2000 | 1.6458 | 1.5729 | 1.7203 |
| GOA | 6.2250 | 5.5250 | 6.1042 | 6.2083 | 6.0156 |
| CDO | 6.3000 | 5.2000 | 6.0625 | 5.7917 | 5.8385 |
| RIME | 3.7750 | 3.9875 | 3.6875 | 3.6667 | 3.7792 |
| SABO | 5.1750 | 4.8750 | 5.5208 | 5.6250 | 5.2989 |

Note. The four component columns contain the archived Friedman ranks of best, worst, standard deviation (Std), and mean final objective values. Average rank is their arithmetic mean; lower values indicate better rank. Rounding of the component ranks can produce a last-digit difference in the displayed average.

|   **(A) CEC2020 F3, D = 10** |   **(B) CEC2020 F6, D = 10** |   **(C) CEC2020 F9, D = 10** |
| --- | --- | --- |
|   **(D) CEC2020 F3, D = 20** |   **(E) CEC2020 F6, D = 20** |   **(F) CEC2020 F9, D = 20** |

**Supplementary Figure S1. Convergence curves for representative benchmark functions.** (A-C) CEC2020 at D = 10; (D-F) CEC2020 at D = 20. Each panel summarizes 30 runs. Columns show multimodal (F3), hybrid (F6), and composition (F9) functions. The CEC2022 panels (G-L) continue on the following page.

|   **(G) CEC2022 F3, D = 10** |   **(H) CEC2022 F6, D = 10** |   **(I) CEC2022 F11, D = 10** |
| --- | --- | --- |
|   **(J) CEC2022 F3, D = 20** |   **(K) CEC2022 F6, D = 20** |   **(L) CEC2022 F11, D = 20** |

**Supplementary Figure S1 (continued). Convergence curves for representative benchmark functions.** (G-I) CEC2022 at D = 10; (J-L) CEC2022 at D = 20. Each panel summarizes 30 runs. Columns show multimodal (F3), hybrid (F6), and composition (F11) functions.

|   **(A) CEC2020 F3, D = 10** |   **(B) CEC2020 F6, D = 10** |   **(C) CEC2020 F9, D = 10** |
| --- | --- | --- |
|   **(D) CEC2020 F3, D = 20** |   **(E) CEC2020 F6, D = 20** |   **(F) CEC2020 F9, D = 20** |
|   **(G) CEC2022 F3, D = 10** |   **(H) CEC2022 F6, D = 10** |   **(I) CEC2022 F11, D = 10** |
|   **(J) CEC2022 F3, D = 20** |   **(K) CEC2022 F6, D = 20** |   **(L) CEC2022 F11, D = 20** |

**Supplementary Figure S2. Final objective-value distributions for representative benchmark functions.** Each panel summarizes 30 runs. (A-C) CEC2020 at D = 10; (D-F) CEC2020 at D = 20; (G-I) CEC2022 at D = 10; (J-L) CEC2022 at D = 20. The function and dimension are identified below each panel.

**Supplementary Figure S3. Arithmetic mean of four Friedman summary ranks.** The component ranks describe best, worst, standard deviation, and mean final objective values (Table S6). Lower average rank is better. The figure summarizes the archived rank results; it is a descriptive average rather than a pooled Friedman analysis or a significance test.

# S2 Robot-level path-planning results

Tables S7-S12 give the robot-level and scenario-level results summarized in Table 3. Total movement steps sum the robot-specific counts, rather than joint replanning cycles. Aggregate path length sums the per-robot average distances. AET includes visualization and pauses, and total fitness sums optimizer-returned costs over replanning calls. Inf denotes nonfinite DWA fitness and is excluded from cost comparisons; it does not itself indicate navigation failure. Main-text Figure 7 visualizes the path lengths in Tables S8 and S11. Main-text Figure 8 presents mean-fitness trajectories without replicate-level uncertainty estimates. The averaging convention for completed runs is unspecified; the curves are interpreted as trajectory trends, without inferring a zero-cost endpoint.

**Supplementary Table S7. Per-robot movement steps in Scenario 1.**

| **Robot** | **SFOA** | **sdSCA** | **SAO** | **EA4eig** | **DWA** | **DCFO** |
| --- | --- | --- | --- | --- | --- | --- |
| Robot1 | 142 | 84 | 96 | 83 | 68 | 81 |
| Robot2 | 166 | 81 | 89 | 90 | 76 | 82 |
| Robot3 | 64 | 33 | 34 | 34 | 35 | 33 |
| Robot4 | 162 | 78 | 90 | 82 | 69 | 75 |
| Robot5 | 92 | 41 | 46 | 46 | 41 | 42 |
| Robot6 | 135 | 64 | 66 | 70 | 64 | 63 |
| Total | 761 | 381 | 421 | 405 | 353 | 376 |

Note. Each value is the movement-step count for the indicated robot and method. Summing robot rows gives total robot movement steps in Table 3; this total is not the number of joint replanning cycles.

**Supplementary Table S8. Average path length (cm) of each robot and method in Scenario 1.**

| **Robot** | **SFOA** | **sdSCA** | **SAO** | **EA4eig** | **DWA** | **DCFO** |
| --- | --- | --- | --- | --- | --- | --- |
| Robot1 | 174.5807 | 100.1103 | 106.4636 | 99.22412 | 101.6 | 96.87224 |
| Robot2 | 198.8019 | 98.80242 | 98.99012 | 98.33742 | 111.6 | 95.35461 |
| Robot3 | 79.9615 | 40.59901 | 39.45341 | 40.55743 | 48.4 | 40.20104 |
| Robot4 | 199.5423 | 95.44923 | 102.985 | 99.82858 | 102.9 | 90.74785 |
| Robot5 | 112.7985 | 49.40704 | 52.3307 | 51.98891 | 60.5 | 49.28698 |
| Robot6 | 166.3799 | 77.20349 | 76.84122 | 79.38814 | 94.5 | 76.47548 |
| Total | 932.0647 | 461.5715 | 477.0641 | 469.3246 | 519.5 | 448.9382 |

Note. Entries are per-robot average path lengths in centimeters. Summing the robot rows gives aggregate path length in Table 3, subject to rounding. Path length is distinct from movement-step count because commanded displacement can vary between steps.

**Supplementary Table S9. Average execution time and total fitness in Scenario 1.**

| **Measure** | **SFOA** | **sdSCA** | **SAO** | **EA4eig** | **DWA** | **DCFO** |
| --- | --- | --- | --- | --- | --- | --- |
| AET (s) | 79.58416 | 40.21232 | 46.43029 | 60.26596 | 1.35E+02 | 54.19103 |
| Total fitness | 3.15E+04 | 1.53E+04 | 1.71E+04 | 1.60E+04 | Inf | 1.49E+04 |

Note. AET is average simulation execution time in seconds, including visualization and pauses; it is not optimizer runtime or control-cycle latency alone. Total fitness sums optimizer-returned cost values over replanning calls. Inf denotes nonfinite DWA fitness, consistent with Table 3. These values are excluded from cost comparisons and do not themselves indicate navigation failure.

**Supplementary Table S10. Per-robot movement steps in Scenario 2.**

| **Robot** | **SFOA** | **sdSCA** | **SAO** | **EA4eig** | **DWA** | **DCFO** |
| --- | --- | --- | --- | --- | --- | --- |
| Robot1 | 129 | 54 | 51 | 50 | 49 | 47 |
| Robot2 | 147 | 57 | 56 | 47 | 35 | 40 |
| Robot3 | 178 | 60 | 69 | 80 | 46 | 48 |
| Robot4 | 91 | 22 | 21 | 20 | 21 | 19 |
| Robot5 | 112 | 30 | 30 | 31 | 29 | 28 |
| Robot6 | 199 | 82 | 95 | 64 | 64 | 66 |
| Robot7 | 86 | 53 | 51 | 45 | 42 | 41 |
| Robot8 | 104 | 42 | 44 | 31 | 28 | 31 |
| Robot9 | 45 | 34 | 23 | 20 | 22 | 22 |
| Robot10 | 91 | 49 | 35 | 35 | 27 | 39 |
| Robot11 | 99 | 38 | 40 | 25 | 24 | 25 |
| Robot12 | 203 | 54 | 53 | 50 | 42 | 46 |
| Total | 1484 | 575 | 568 | 498 | 429 | 452 |

Note. Each value is the movement-step count for the indicated robot and method. Summing robot rows gives total robot movement steps in Table 3; this total is not the number of joint replanning cycles.

**Supplementary Table S11. Average path length (cm) of each robot and method in Scenario 2.**

| **Robot** | **SFOA** | **sdSCA** | **SAO** | **EA4eig** | **DWA** | **DCFO** |
| --- | --- | --- | --- | --- | --- | --- |
| Robot1 | 157.3911 | 65.45156 | 56.9385 | 60.46888 | 71.3 | 56.59856 |
| Robot2 | 182.5159 | 65.85011 | 61.54699 | 56.33114 | 50.1 | 48.35103 |
| Robot3 | 220.5002 | 74.3121 | 77.91525 | 96.2252 | 68.4 | 59.40608 |
| Robot4 | 111.7973 | 26.03487 | 23.83605 | 24.93845 | 30.8 | 23.25285 |
| Robot5 | 140.4047 | 36.60731 | 34.89791 | 37.3673 | 42.4 | 34.83387 |
| Robot6 | 248.0943 | 95.95157 | 104.656 | 77.9885 | 93.8 | 79.75926 |
| Robot7 | 104.979 | 64.78169 | 58.08748 | 53.77568 | 62.1 | 49.99447 |
| Robot8 | 128.6594 | 50.05911 | 47.46415 | 37.76411 | 38 | 37.5833 |
| Robot9 | 55.54469 | 41.67457 | 25.09414 | 23.83429 | 31.5 | 26.11494 |
| Robot10 | 110.372 | 58.46681 | 39.54547 | 39.68661 | 39.8 | 46.69388 |
| Robot11 | 123.4281 | 45.47656 | 44.0932 | 30.70011 | 35.3 | 31.23487 |
| Robot12 | 250.0215 | 66.06813 | 58.82568 | 59.45838 | 61.7 | 55.04057 |
| Total | 1833.708 | 690.7344 | 632.9008 | 598.5387 | 625.2 | 548.8637 |

Note. Entries are per-robot average path lengths in centimeters. Summing the robot rows gives aggregate path length in Table 3, subject to rounding. Path length is distinct from movement-step count because commanded displacement can vary between steps.

**Supplementary Table S12. Average execution time and total fitness in Scenario 2.**

| **Measure** | **SFOA** | **sdSCA** | **SAO** | **EA4eig** | **DWA** | **DCFO** |
| --- | --- | --- | --- | --- | --- | --- |
| AET (s) | 1.96E+02 | 77.02411 | 87.00167 | 90.68558 | 313.5523 | 74.75454 |
| Total fitness | 9.06E+04 | 1.47E+04 | 3.46E+04 | 1.31E+04 | Inf | 1.14E+04 |

Note. AET is average simulation execution time in seconds, including visualization and pauses; it is not optimizer runtime or control-cycle latency alone. Total fitness sums optimizer-returned cost values over replanning calls. Inf denotes nonfinite DWA fitness, consistent with Table 3. These values are excluded from cost comparisons and do not themselves indicate navigation failure.
